# Development of examination objectives based on nursing competency for the Korean Nursing Licensing Examination: a validity study

**DOI:** 10.3352/jeehp.2022.19.19

**Published:** 2022-08-22

**Authors:** Sujin Shin, Gwang Suk Kim, Jun-Ah Song, Inyoung Lee

**Affiliations:** 1 College of Nursing, Ewha Womans University, Seoul, Korea; 2 College of Nursing, Mo-Im Kim Nursing Research Institute, Yonsei University, Seoul, Korea; 3 College of Nursing, BK21 FOUR R&E Center for Learning Health Systems, Korea University, Seoul, Korea; Hallym University, Korea

**Keywords:** Nursing licensure, Nurse’s role, Nursing education, Republic of Korea

## Abstract

**Purpose:**

This study aimed to develop the examination objectives based on nursing competency of the Korean Nursing Licensing Examination.

**Methods:**

This is a validity study to develop the examination objectives based on nursing competency. Data were collected in December 2021. We reviewed the literature related to changing nurse roles and on the learning objectives for the Korea Medical Licensing Examination and other health personnel licensing examinations. Thereafter, we created a draft of the nursing problems list for examination objectives based on the literature review, and the content validity was evaluated by experts. A final draft of the examination objectives is presented and discussed.

**Results:**

A total of 4 domains, 12 classes, and 85 nursing problems for the Korean Nursing Licensing Examination were developed. They included the essentials of objectives, related factors, evaluation goals, related activity statements, related clients, related settings, and specific outcomes.

**Conclusion:**

This study developed a draft of the examination objectives based on clinical competency that were related to the clinical situations of nurses and comprised appropriate test items for the licensing examination. Above results may be able to provide fundamental data for item development that reflects future nursing practices.

## Introduction

### Background/rationale

The Korean Nursing Licensing Examination aims to verify the minimum competency required to perform the clinical practice as a new nurse. However, the items for the Korean Nursing Licensing Examination were written according to the subject-centered learning objectives of the nursing college curriculum [1]. Limitations have been raised in the evaluation of the minimum competency required to perform as a registered nurse [2]. To overcome these limitations, many studies have been conducted to reflect clinical practice in the exams such as job analysis and defining the minimum competency of new nurses, and linking this with learning objectives [3-5], Kim et al. [6] in 2018 presented an activity statement suitable for the Korean clinical situation based on the previous study that was analyzed the duties of new nurses, linked them with learning objectives, and defined the minimum competency of new nurses [5]. These studies provide a basis for proposing an integrated model for the Korean Nursing Licensing Examination.

Although previous studies have suggested the basis for verifying the competence of new nurses to be evaluated in the Korean Nursing Licensing Examination, there is a limit to applying this result to the implementation of the Korean Nursing Licensing Examination. The examination objectives for the licensing examination should consider the competency for expansion of nurses’ workplaces and their expanding roles according to changes in the medical environment, such as the increase in the elderly population, the pandemic of infectious disease, and the development of the technology. In medical education, the objectives for the Korean Medical Licensing Examination of theoretical knowledge and practicum have been developed and used to develop items for licensure examination [7,8]. The development of examination objectives for the Korean Nursing Licensing Examination is required as an essential research task that can no longer be belayed.

Therefore, in this study, we developed a draft of the examination objectives for the Korean Nursing Licensing Examination that reflects clinical practice to evaluate the minimum competency of new nurses and presented the specific contents of each objective for the examinations.

### Objectives

This study aimed to develop the examination objectives for the Korean Nursing Licensing Examination, verify its content validity, and present the contents of each objective for the examinations.

## Methods

### Ethics statement

To confirm the validity of the content, information on the examination objectives developed by the experts and explanations of the research objectives were provided. The validity of the experts was not exposed to personal information at the time of the survey and was carried out in terms of enlargement. Therefore, responding to the inquiry was regarded as prior consent.

### Study design

It is a validity study. This was conducted in the following phases: (1) review and analysis of related literature, (2) development of a draft of examination objectives, (3) verifying the contents validity by experts, (4) expert consulting, and (5) a public hearing from related experts.

### Setting

A validity survey was conducted with experts from 17th to 29th, December 2021 to select the final list to be developed as the objective for examination. The experts were selected from nursing education and clinical professionals.

### Participants

Experts for validation were selected from the nursing educator and clinical nurses. The inclusion criteria were nursing college professors or clinical nurses who had experience in developing or reviewing items in the Korean Nursing Licensing Examination and educational nurses participating in newly graduated nurses’ education. A total of 50 individuals (30 professors and 20 clinical nurses) participated in the survey. The average length of the education experience of professors was 20.3±7.7 years, and the average length of clinical experience of clinical nurses was 18.8±11.4 years (Table 1).

### Variables and measurement

We reviewed the current Korean Nursing Licensing Examination and the trend of expanding the role of nurses. We collected data from 50 experts on the content validity index (CVI) that developed a draft of the examination objectives for the nursing licensing examination based on the literature review and its analysis (Dataset 1). Content validity was assessed by a group of expert nurses. The importance and possibility of item development in the selected nursing problems for content validity were evaluated using a 4-point Likert scale. Importance refers to a nursing problem that occurs frequently in the clinical situation of a new nurse or can seriously change a client's health status if appropriate nursing intervention is not performed. Possibility of item development refers to whether it is appropriate for the content to be evaluated in the nursing licensing examination. As the next step in evaluating CVI, the contents were reviewed once again through an open hearing. We present and discuss the final draft of the examination objectives of the nursing licensing examination.

### Study size

The size of the expert group for verifying content validity varies from 5 to 10 [9] or 20 [10], but theoretical and practical preparations for the concept to be measured should be considered important [11]. To collect the diverse opinions of educational and clinical experts, we included more than 20 people in each field.

### Statistical methods

We calculated the content validity index (CVI), which is the percentage of experts who answered 3 or 4 points out of the scores answered on a 4-point scale for each question by a group of experts. The CVI (average content validity index for scale, S-CVI/Ave) for all measurement tools was calculated by dividing the I-CVI by the number of questions.

## Results

### Definitions and components of the examination objectives for the Korean Nursing Licensing Examination

As a basic analysis of the development of the draft objectives for examination, we reviewed the literature related to the licensing exam for nurses and examination objectives for other health personnel. Based on the literature review, the examination objectives for the Korean Nursing licensing examination were defined as describing the minimum competence that nurses should have and orienting on the job situations that may occur. In addition, the contents to be included in the objectives were determined as the essentials or rationale of each evaluation objective, related factors, evaluation goals, related activity statements, related clients, related settings, and specific outcomes.

### Selection of nursing problem list for developing examination objectives

An initial list of examination objectives was developed based on a literature review of the nursing licensing examination and nursing problems of the North American Nursing Diagnosis Association International (NANDA-I) [12], which is a standardized nursing classification system. A total of 13 domains, 45 classes, and 171 nursing problems were derived from the list of problems faced by new nurses. A content validity survey was conducted with a group of experts to determine the priority of the derived nursing problems. The validity of importance in practice was 0.52 to 1.00 and the average was 0.86; the validity of possibility for the items was 0.46 to 1.00 and the average was 0.84. Of the 171 nursing problems, 130 had a CVI ≥0.75 in both importance and possibility validity. Based on the results of the validation by the experts, a total of 13 domains, 45 classes, and 88 nursing problems were selected to develop the examination objectives (Table 2). Raw responses data of participants are available from Dataset 1.

### Development of the draft examination objectives for the Korean Nursing Licensing Examination

For the final list of examination objectives for the Korean Nursing Licensing Examination, expert consultation was conducted by 2 nursing college professors and 2 clinical nurses with experience in writing and reviewing items for the Korean Nursing Licensing Examination. After consulting experts, 3 similar nursing problems were integrated, and finally, 4 domains, 12 classes, and 85 nursing problems were selected. The contents were derived from previous studies related to nursing problems and minimum competency. The examination objectives were comprised of the necessity or rationale of each examination objective, related factors, evaluation goals, related activity statements, related clients (individual, family, community/population group), related settings (acute, chronic disease management, wellness, prevention, regenerative/restorative, hospice/palliative), and specific outcomes.

## Discussion

### Interpretation

In this study, we explored the significance of the licensing examination for nurses and the importance of objectives for examinations, defined examination objectives for nursing licensing examinations, and developed examination objectives for the Korean Nursing Licensing Examination. Since they were developed based on the minimum competency of nurses and clinical situations, there are no boundaries between nursing subjects. In this study, based on NANDA-I, a globally standardized nursing classification system, nursing problems experienced by new nurses in the workplace are set as clinical situations, and the importance of clinical situations is judged based on the common training contents of new nurses, and the clinical situation of nurses and the minimum competency were linked.

The content validity by the experts in selecting the list of the final objectives for examination verified the importance in practice and the possibility of the items in the licensing examination. As a result, we removed the list of objectives with low practical importance and low possibility. Although the CVI for both importance and possibility was low, there were also items with an average expert validity score of 3 or higher.

Items that are important in the practice of new nurses but are inappropriate for items in the licensing exams include “impaired environmental comfort,” “impaired parenting,” “stress overload,” “moral distress,” “disturbed personal identity” and “situational low self-esteem.” These are important in practice and should be included in education; however, they can be evaluated as inappropriate as items. Contrarily, the nursing problems such as “dysfunctional family processes,” “sexual dysfunction,” and “ineffective denial” are not problems that new nurses often encounter or have fatal consequences if they do not apply appropriate intervention. However, these problems can be dealt with like items for the licensing examination. As such, for the development of the examination objectives of the Korean Nursing Licensing Examination, there may be problems that are important in the practice of new nurses but are not appropriate for the examination. Therefore, the objectives for the examination are related to major nursing problems rather than including all job situations, and it is necessary to include items that are valid as exam items.

There was a study on the standard setting of the Korean Nursing Licensing Examination using Angoff method [13]. For this high-stakes examination, the classification of nursing problems is also essential to set the cut score because the performance level description of the overall exam and each subject is the basic step.

### Limitations

A total of 85 nursing problems were selected as examination objectives for the Korean Nursing Licensing Examination based on a literature review and expert consultation. Objectives for the examination of the selected nursing problems were developed and comprised rationales, related factors, evaluation goals, related activity statements, related goals, related clients, related settings, and specific outcomes. However, the validation of the specific contents of the objectives has not been evaluated.

### Generalizability

Results in this study may be able to be applied to other countries because the nursing competency is almost the same in all countries.

### Suggestions

The developed objectives for the examination presented relevant activity statements and outcomes; however, there is a limit to direct application to develop items for evaluation. Therefore, through further research, it is necessary to organize relevant activity statements into core competencies and verify their validity by modifying them to become guidelines for item development. Based on the objectives of the examination, it is necessary to review the linkage with the existing Korean Nursing Licensing Examination test items and investigate the basis for developing it as an objective for examination that can be used as a unit of items.

In addition, it must be revised and developed so that it can supply objectives for the examination of integrated items without boundaries of nursing subjects. Furthermore, it is necessary to develop an examination objective that can extend the applicability of appropriate clinical judgment and nursing processes to major nursing situations faced by new nurses.

### Conclusion

This study developed the examination objectives of the Korean Nursing Licensing Examination to reflect the practical situation of nurses. The developed objectives are related to the job situation of new nurses and consist of problems that are appropriate for evaluation during the exam. This provides a basis for the practical competency evaluation of nurses. Therefore, this study provides fundamental data for future test plans centered on practical competency.

## Figures and Tables

**Table 1.** The characteristics of participants (N=50)

Characteristic	No. (%)
Age (yr)	
≤40	9 (18.0)
41–50	16 (32.0)
51–60	19 (38.0)
>60	6 (12.0)
Location of worksite	
Seoul	17 (34.0)
Incheon, Gyeonggi, Gangwon	8 (16.0)
Gwangju, Jeolla	4 (8.0)
Busan, Ulsan, Daegu, Gyengsan	11 (22.0)
Daejeon, Chungcheong	10 (20.0)
Affiliation	
Nursing education institution	30 (60.0)
Clinical setting	
Hospital	16 (32.0)
Public health	2 (4.0)
Occupational	1 (2.0)
Long-term care facilities	1 (2.0)
Education	
Baccaluareate	7 (14.0)
Master	5 (10.0)
Dorctoral	38 (76.0)
Experience in the Korean Nursing Licensing Examination	
No	19 (38.0)
Yes	
Item development^a)^	22 (44.0)
Test consultaion^a)^	18 (38.0)
Item review^a)^	20 (40.0)

a) Multiple respomses.

**Table 2.** Content validity index for nursing problems

Domain/class	Nursing problems	Importance		Possibility	
		CVI^a)^	Mean	CVI^a)^	Mean
1. Health promotion					
Health awareness	Sedentary lifestyle	0.84	3.06	0.78	2.90
	Readiness for enhanced health literacy	0.74	3.00	0.68	2.82
Health management	Frail elderly syndrome	0.80	3.22	0.82	3.18
	Deficient community health	0.72	2.88	0.74	2.94
	Risk-prone health behavior	0.82	3.28	0.86	3.32
	Ineffective health maintenance behaviors	0.84	3.32	0.84	3.30
	Ineffective family health self-management	0.78	2.98	0.78	3.02
	Ineffective home maintenance behaviors	0.66	2.70	0.58	2.69
	Ineffective protection	0.68	2.82	0.60	2.88
2. Growth/development					
Growth	Growth	0.88	3.42	0.84	3.42
Development	Delayed child development	0.90	3.46	0.94	3.54
	Delayed infant motor development	0.86	3.34	0.88	3.36
3. Nutrition					
Ingestion	Imbalanced nutrition	0.98	3.70	0.96	3.72
	Insufficient breast milk production	0.78	3.10	0.80	3.08
	Ineffective breastfeeding	0.84	3.24	0.86	3.28
	Obesity	0.82	3.34	0.82	3.38
	Overweight	0.82	3.18	0.80	3.24
	Impaired swallowing	0.94	3.68	0.94	3.68
Digestion	Impaired digestion	0.96	3.68	0.92	3.62
Absorption	Impared absorption	0.96	3.62	0.92	3.54
Metabolism	Risk for unstable blood glucose level	1.00	3.94	1.00	3.90
	Hyperbilirubinemia	0.92	3.62	0.92	3.54
	Risk for impaired liver function	0.98	3.82	0.98	3.78
	Risk for metabolic syndrome	1.00	3.84	1.00	3.86
Hydration	Risk for electrolyte imbalance	1.00	3.94	1.00	3.94
	Risk for imbalanced fluid volume	1.00	3.88	0.98	3.84
	Deficient fluid volume	1.00	3.88	0.98	3.84
	Excess fluid volume	0.98	3.82	0.98	3.82
4. Elimination and exchange					
Urinary function	Disability-associated urinary incontinence	0.84	3.24	0.82	3.24
	Urinary incontinence	0.86	3.44	0.92	3.54
	Impaired urinary elimination	1.00	3.78	1.00	3.80
	Urinary retention	0.98	3.70	0.96	3.66
Gastrointestinal function	Constipation	0.90	3.50	0.88	3.48
	Bowel continence	0.84	3.30	0.82	3.26
	Diarrhea	0.96	3.70	0.98	3.68
	Impaired gastrointestinal motility	0.96	3.68	0.96	3.70
Integumentary function	Impaired integumentary function	0.96	3.62	0.98	3.64
Respiratory function	Impaired gas exchange	1.00	3.88	1.00	3.88
5. Activity/rest					
Sleep/rest	Sleep disturbance	0.88	3.30	0.84	3.24
Activity/exercise	Decreased activity tolerance	0.86	3.26	0.86	3.26
	Risk for disuse syndrome	0.82	3.14	0.78	3.10
	Impaired mobility	0.92	3.46	0.90	3.44
	Impaired sitting	0.82	3.08	0.80	3.06
	Impaired standing	0.80	3.10	0.80	3.08
	Impaired walking	0.88	3.42	0.92	3.44
Energy balance	Imbalanced energy field	0.66	2.78	0.62	2.68
	Fatigue	0.80	3.18	0.80	3.18
	Wandering (dementia)	0.68	2.98	0.72	3.02
Cardiovascular/pulmonary responses	Ineffective breathing pattern	0.98	3.90	1.00	3.92
	Decreased cardiac output	1.00	3.94	1.00	3.94
	Risk for impaired cardiovascular function	1.00	3.88	1.00	3.88
	Ineffective lymphedema self-management	0.92	3.50	0.90	3.50
	Impaired spontaneous ventilation	0.98	3.82	0.98	3.80
	Risk for unstable blood pressure	1.00	3.88	1.00	3.88
	Risk for thrombosis	1.00	3.90	1.00	3.88
	Risk for decreased cardiac tissue perfusion	1.00	3.86	0.98	3.84
	Risk for ineffective cerebral tissue perfusion	1.00	3.90	1.00	3.88
	Ineffective peripheral tissue perfusion	0.98	3.82	1.00	3.82
	Dysfunctional ventilatory weaning response	0.94	3.58	0.94	3.56
Self-care	Activity of daily living self-care deficit	0.86	3.34	0.86	3.34
	Self-neglect	0.60	2.76	0.60	2.74
6. Perception/cognition					
Attention	Unilateral neglect	0.96	3.56	0.94	3.52
Orientation	Impaired orientation	0.98	3.74	0.96	3.74
Sensation/perception	Impaired sensation/perception	0.98	3.76	0.96	3.76
Cognition	Acute confusion	0.96	3.72	0.96	3.74
	Chronic confusion	0.92	3.46	0.92	3.50
	Labile emotional control	0.82	3.38	0.82	3.38
	Ineffective impulse control	0.80	3.28	0.78	3.28
	Deficient knowledge	0.94	3.58	0.90	3.46
	Impaired memory	0.84	3.34	0.82	3.28
	Disturbed thought process	0.84	3.26	0.82	3.22
Communication	Impaired verbal communication	0.86	3.46	0.88	3.50
7. Safety/protection					
Infection	Risk for infection	1.00	3.92	1.00	3.94
	Risk for surgical site infection	0.94	3.72	0.94	3.70
Physical injury	Ineffective airway clearance	1.00	3.92	1.00	3.92
	Risk for aspiration	1.00	3.92	1.00	3.92
	Risk for bleeding	1.00	3.94	1.00	3.94
	Impaired dentition	0.62	2.58	0.52	2.44
	Risk for dry eye	0.78	3.12	0.74	3.04
	Ineffective dry eye self-management	0.74	2.98	0.74	2.92
	Risk for dry mouth	0.80	3.20	0.80	3.14
	Risk fall	1.00	3.82	1.00	3.84
	Risk for injury	0.92	3.60	0.90	3.60
	Risk for corneal injury	0.84	3.20	0.82	3.20
	Nipple-areolar complex injury	0.72	2.86	0.66	2.78
	Risk for urinary tract injury	0.90	3.52	0.90	3.52
	Risk for perioperative positioning injury	0.90	3.40	0.88	3.34
	Risk for thermal injury	0.88	3.50	0.92	3.54
	Impaired oral mucous membrane integrity	0.88	3.44	0.86	3.38
	Risk for peripheral neurovascular dysfunction	0.94	3.64	0.96	3.60
	Risk for physical trauma	0.94	3.66	0.90	3.60
	Risk for vascular trauma	0.94	3.60	0.94	3.60
	Adult pressure injury	0.98	3.82	0.98	3.80
	Child pressure injury	0.88	3.48	0.82	3.36
	Neonatal pressure injury	0.88	3.44	0.80	3.28
	Risk for shock	1.00	3.90	1.00	3.90
	Impaired skin integrity	0.94	3.74	0.98	3.74
	Risk for sudden infant death	0.90	3.54	0.94	3.54
	Risk for suffocation	0.92	3.72	0.92	3.66
	Delayed surgical recovery	0.96	3.52	0.96	3.50
	Impaired tissue integrity	0.96	3.60	0.94	3.56
Violence	Risk for female genital mutilation	0.82	3.28	0.82	3.24
	Risk for other-directed violence	0.80	3.24	0.76	3.12
	Risk for self-directed violence	0.74	3.14	0.74	3.08
	Self-mutilation	0.84	3.30	0.82	3.28
	Risk for suicidal behavior	0.92	3.58	0.90	3.54
Environmental hazards	Contamination	0.86	3.28	0.80	3.20
	Risk for occupational injury	0.82	3.20	0.78	3.14
	Risk for poisoning	0.90	3.40	0.86	3.32
Defensive processes	Risk for adverse reaction to iodinated contrast media	0.94	3.36	0.92	3.36
	Risk for allergy reaction	1.00	3.70	1.00	3.70
	Risk for latex allergy reaction	0.64	2.80	0.62	2.72
Thermoregulation	Hyperthermia	1.00	3.84	1.00	3.82
	Hypothermia	0.98	3.80	0.98	3.78
	Neonatal hypothermia	0.92	3.62	0.92	3.60
	Risk for perioperative hypothermia	0.90	3.30	0.90	3.34
	Ineffective thermoregula	0.96	3.64	0.96	3.66
8. Comfort					
Physical comfort	Impaired physical comfort	0.94	3.54	0.94	3.54
	Nausea	1.00	3.64	1.00	3.64
	Acute pain	1.00	3.88	1.00	3.86
	Chronic pain	1.00	3.72	1.00	3.68
	Labor pain	0.90	3.50	0.90	3.48
Environmental comfort	Impaired environmental comfort	0.78	3.08	0.70	2.90
Social comfort	Impaired social comfort	0.74	2.96	0.70	2.88
9. Role relationship					
Caregiving roles	Impaired parenting	0.80	3.12	0.84	3.14
	Caregiver role strain (burden)	0.80	3.08	0.80	3.02
Family relationships	Risk for impaired attachment	0.78	3.12	0.80	3.10
	Disturbed family identity syndrome	0.68	2.80	0.66	2.82
	Dysfunctional family processes	0.70	2.90	0.76	2.96
Role performance	Ineffective relationship	0.68	2.88	0.66	2.86
	Parental role conflict	0.78	3.02	0.74	2.96
	Ineffective role performance	0.74	3.00	0.74	2.96
	Impaired social interaction	0.72	2.94	0.74	2.92
10. Sexuality					
Sexual identity	Sexual identity	0.70	2.78	0.74	2.82
Sexual function	Sexual dysfunction	0.74	3.00	0.78	2.98
	Ineffective sexuality pattern	0.76	2.92	0.76	2.88
Reproduction	Ineffective childbearing process	0.90	3.38	0.90	3.42
	Risk for disturbed maternal-fetal dyad	0.84	3.28	0.84	3.28
11. Coping/stress tolerance					
Post-trauma responses	Risk for complicated immigration transition	0.60	2.60	0.50	2.52
	Post-trauma syndrome	0.86	3.36	0.90	3.36
	Rape-trauma syndrome	0.82	3.22	0.80	3.14
	Relocation stress syndrome	0.78	3.10	0.76	3.06
Coping responses	Ineffective activity planning	0.66	2.90	0.68	2.92
	Anxiety	0.98	3.60	0.96	3.56
	Ineffective coping	0.92	3.50	0.88	3.38
	Ineffective community coping	0.74	3.08	0.66	3.00
	Compromised family coping	0.72	3.04	0.68	2.94
	Death anxiety	0.88	3.42	0.84	3.28
	Ineffective denial	0.72	3.04	0.76	2.98
	Fear	0.80	3.26	0.78	3.14
	Maladaptive grieving	0.74	3.08	0.72	2.96
	Impaired mood regulation	0.78	3.18	0.76	3.10
	Powerlessness	0.80	3.18	0.76	3.06
	Impaired resilience	0.72	3.04	0.70	2.94
	Chronic sorrow	0.70	2.94	0.68	2.90
	Stress overload	0.76	3.18	0.72	3.10
Neurobehavioral stress	Neurobehavioral stress	0.86	3.30	0.88	3.30
	Autonomic dysreflexia	0.82	3.12	0.82	3.12
	Neonatal abstinence syndrome	0.74	2.98	0.70	2.94
	Disorganized infant behavior	0.80	3.00	0.80	2.96
12. Life principles					
Value/belief/action congruence	Decisional conflict	0.84	3.22	0.84	3.12
	Impaired emancipated decision-making	0.72	2.98	0.66	2.84
	Moral distress	0.76	3.02	0.60	2.82
	Impaired religiosi	0.52	2.54	0.46	2.42
	Spiritual distress	0.60	2.64	0.52	2.52
13. Self-perception					
Self-concept	Hopelessness	0.78	3.10	0.62	2.86
	Risk for compromised human dignity	0.72	2.96	0.68	2.86
	Disturbed personal identity	0.76	3.02	0.74	2.94
Self-esteem	Chronic low self-esteem	0.74	3.04	0.68	2.86
	Situational low self-esteem	0.76	3.08	0.68	2.88
Body image	Disturbed body image	0.82	3.18	0.78	3.06

a) CVI, content validity index.

## Footnotes

**Data availability**
Data files are available from Harvard Dataverse: https://doi.org/10.7910/DVN/PAAQ53 Dataset 1. Raw response data of participants.

## Supplementary materials

Supplement 1. Audio recording of the abstract.
